# Precision public health in schools enabled by wastewater surveillance: A case study of COVID-19 in an Upstate New York middle-high school campus during the 2021–2022 academic year

**DOI:** 10.1371/journal.pgph.0001803

**Published:** 2024-01-10

**Authors:** Haley Kappus-Kron, Dana Ahmad Chatila, Ainsley Mabel MacLachlan, Nicole Pulido, Nan Yang, David A. Larsen

**Affiliations:** 1 Center for Environmental Health, New York State Department of Health, Albany, New York, United States of America; 2 CDC Foundation, Atlanta, Georgia, United States of America; 3 Department of Public Health, Syracuse University, Syracuse, New York, United States of America; New York University Grossman School of Medicine, UNITED STATES

## Abstract

Wastewater surveillance provides a cost-effective and non-invasive way to gain an understanding of infectious disease transmission including for COVID-19. We analyzed wastewater samples from one school site in Jefferson County, New York during the 2021–2022 school year. We tested for SARS-CoV-2 RNA once weekly and compared those results with the clinical COVID-19 cases in the school. The amount of SARS-CoV-2 RNA correlated with the number of incident COVID-19 cases, with the best correlation being one day lead time between the wastewater sample and the number of COVID-19 cases. The sensitivity and positive predictive value of wastewater surveillance to correctly identify any COVID-19 cases up to 7 days after a wastewater sample collection ranged from 82–100% and 59–78% respectively, depending upon the amount of SARS-CoV-2 RNA in the sample. The specificity and negative predictive value of wastewater surveillance to correctly identify when the school was without a case of COVID-19 ranged from 67–78% and 70–80%, respectively, depending upon the amount of SARS-CoV-2 RNA in the sample. The lead time observed in this study suggests that transmission might occur within a school before SARS-CoV-2 is identified in wastewater. However, wastewater surveillance should still be considered as a potential means of understanding school-level COVID-19 trends and is a way to enable precision public health approaches tailored to the epidemiologic situation in an individual school.

## 1. Introduction

In response to the COVID-19 pandemic, Governor Andrew Cuomo of New York State issued an executive order closing all schools on March 18, 2020 for a period of two weeks [1]. That school closure was then continued through the end of the school year, before some schools returned to at least partial in-person schooling in the fall of 2020. In-person education was limited across New York state during the 2020–21 academic year, as many school districts restricted in-person instruction to reduced numbers of students in classrooms. Remote learning placed a significant strain on students, caregivers, and educators. Multiple studies suggest that students lost significant learning [2], with the greatest losses being among lower-income students. COVID-19 infection has been linked to decreased cognitive capacity and impaired cognitive development [3–5], and it is important to note that mass COVID-19 infection may be at least partly responsible for this learning slide. The school closures alongside the flatten the curve campaign had their desired effect. Millions of lives were saved as people across the globe limited their social connections and simply stayed home [6, 7]. The interventions bought necessary time for vaccine development and distribution. By the 2021–22 academic year most schools were primarily in person once again.

The school closures, which were also put into place across multiple countries, were implemented before any COVID-19 cases had been observed in the majority of schools. Although a necessary response to the overflowing hospitals and morgues in New York City and other large metropolitan areas given the state of infectious disease surveillance systems at the time, mass school closure was the opposite of precision public health. Given the detrimental effects of school closures, are more precise public health interventions possible [8, 9]. With their dense and intermingling populations, schools play an important role as key settings in regard to infectious disease and can act as transmission accelerators. Students and especially young children often have poor hygiene skills allowing for respiratory infections to spread quickly. Children from multiple households congregate together, allowing for wide dispersion of any infectious diseases present. Even before COVID-19, brief school closures had been utilized to disrupt influenza transmission [10–13].

Precision public health interventions for infectious diseases require strong infectious disease surveillance. For example, prior to vaccine availability, allowing schools to remain open in communities without SARS-CoV-2 transmission (precision school closures) requires infectious disease surveillance capable of both 1) confirming that SARS-CoV-2 transmission is absent in the community and 2) alerting to the arrival of SARS-CoV-2 transmission in the community. Infectious disease surveillance systems relying solely on clinical cases are wholly inadequate for either of these requirements. Clinical cases are only the tip of the iceberg in terms of disease transmission–leakage in the surveillance system can occur with asymptomatic cases, cases that do not seek treatment, cases without access to health care, and a lack of diagnostic capacity. Because of these leakages, SARS-CoV-2 like many other infectious diseases can spread cryptically in a community before they are detected through clinical cases.

Wastewater surveillance for COVID-19 has shown the capacity to confirm communities [14] and university dormitories [15] as free from SARS-CoV-2 transmission, as well as provide early indication before COVID-19 cases are identified [16]. Herein we explore the potential for wastewater surveillance to be deployed at the school level using COVID-19 as a case study, and specifically estimate the sensitivity, specificity, positive predictive value (PPV), and negative predictive (NPV) value of using wastewater surveillance to identify incident COVID-19 cases in a school setting.

## 2. Methods

### 2.1 Setting

The Thousand Islands Central School District (TISCD) is located in Jefferson County, New York and contains four schools including two elementary schools, a middle school, and a high school. The middle school and high school share a campus (Sand Bay Thousand Islands) serving 600 students with an additional 115 faculty and staff. The school district serves a community population of 7,000 residents, with a large increase of tourists during the summer months. The area includes the towns of Clayton and Cape Vincent as well as Roseire, St. Lawrence, Clayton Center, and the hamlets of Depauville and Fisher’s Landing. The TISCD halted in-person activities and school at the start of the COVID-19 pandemic in March 2020, along with all public schools in New York State. The TISCD returned to in-person learning in November 2021.

### 2.2 COVID-mitigation policies

The middle-high school had various policies in place to mitigate COVID-19 cases during the 2021–22 academic year. Many of the policies fall under social distancing guidelines such as limiting the number of students in classrooms, buses, and cafeterias to maintain a 6-foot distance at all times. They used nontraditional rooms for meals such as their stage and conference rooms. Everyone on school property was required to wear a mask at all times throughout the 2021–2022 academic year. The school had testing and take-home tests available for anyone at the district at all times but did not have a systematic testing plan in place. Reporting of positive at-home COVID-19 tests or away-from-school clinical diagnoses to the school nurse was encouraged for contact tracing purposes and the school ensured easy access to rapid diagnostic tests allowing testing for school children and family members. Quarantine and isolation of students and staff changed throughout the year in accordance with the most recent New York State guidelines.

### 2.3 Wastewater data

Wastewater surveillance was a complementary measure for the TICSD re-opening plan and wastewater sampling began in November 2021. The local health department and school administration wanted to understand trends in COVID-19 transmission. The middle-high school campus has a single main sewage line that serves the entirety of the campus. The line sampled only serves the middle and high school campus and excludes any community structures. Thousand Islands School District personnel sampled effluent wastewater from the campus weekly. They pulled 8-hour composite samples (morning to evening) with 5-minute intervals using homemade samplers built from peristaltic pumps [17]. Samples were kept on wet ice and shipped overnight to Quadrant Biosciences located in Syracuse, New York. Quadrant biosciences analyzed wastewater using ultracentrifugation through a sucrose cushion as described by Wilder et al. [18]. In brief, SARS-CoV-2 RNA was quantified using RT-qPCR of the IP2 and IP4 combined primers and cross assembly phage (crAssphage) [19, 20] was also quantified as a quality control measure. Tests were run in triplicate, and we categorized results into one of three categories: not detected, detected but below the limit of quantification (Detected, LOQ), and quantifiable (Quantifiable detection). Not detected is defined as no SARS-CoV-2 RNA detected in any of the three PCR runs. Detected but below the limit of quantification is whenSARS-CoV-2 was detected in at least one of the PCR runs but not in all three above the 5 copies per mL limit of quantification. Samples were quantifiable when all 3 of the PCR runs were above the limit of quantification of 5 genome copies per mL. We further standardize the wastewater testing result as the log-transformed copies of SARS-CoV-2 detected divided by the log-transformed copies of crAssphage detected.

### 2.4 Clinical case data

Throughout the pandemic, incident clinical cases of COVID-19 were recorded by district administrators for contact tracing purposes. These incident cases were reported by the school nurse from testing symptomatic children, by caregivers testing children at home or a doctor’s office (both asymptomatic and symptomatic testing), or by faculty and staff reporting positive results from their own tests (both asymptomatic and symptomatic testing). Clinical COVID-19 cases diagnosed on weekends, holidays, and snow days were included in the the next reporting day in files kept by district administrators. The clinical cases are the cumulation of all reported tests from school personnel and students, whether tests were taken at home or in the clinical setting and including both rapid diagnostic tests and PCR diagnosis. We aggregated daily weekday incident counts between November 4, 2021, and the last day of school on June 16, 2022. We then smooth the case data by taking a seven-day average to account for any potential weekend, snow-day, or holiday heaping.

### 2.5 Data analysis

We classified SARS-CoV-2 RNA in wastewater results as non-detectable, detectable but below the level of quantification, or quantifiable. We then estimated the sensitivity, specificity, NPV, and PPV of the classified wastewater surveillance results to indicate at least one clinical COVID-19 case in schools with varying lead times. We defined a true positive as a COVID-19-positive wastewater surveillance result either detected or quantifiable that coincided with at least one clinical COVID-19 case. We defined a false positive as a COVID-19 positive wastewater surveillance results either detected or quantifiable that coincided with no clinical COVID-19 cases. We define a true negative as a COVID-19 negative wastewater surveillance results either not detected or below the limit of quantification that coincided with no COVID-19 cases. We define a false negative as a COVID-19 negative wastewater surveillance result either not detected or below the limit of quantification that coincided with at least one clinical COVID-19 case. We calculated the sensitivity, specificity, NPV, and PPV with their respective 95% confidence interval with the following equations:

**Table:** 

Eq 1: Sensitivity	$Sensitivity=\frac{true\;positive}{true\;positive+false\;negative}$
Eq 2: Specificity	$Specificity=\frac{true\;negative}{true\;negative+false\;positive}$
Eq 3: Positive Predictive Value (PPV)	$PPV=\frac{true\;positive}{true\;positive+false\;positive}$
Eq 4: Negative Predictive Value (NPV)	$NPV=\frac{true\;negative}{true\;negative+false\;negative}$
Eq 5: Confidence interval wherein the estimate is sensitivity, specificity, PPV, or NPV	$95\%\;CI=estimate\pm 1.96\times {SE}_{proportion}$
Eq 6: Standard error (SE) of the proportion	${SE}_{proportion}=\sqrt{\frac{estimate(1-estimate)}{n}}$

We further assessed the correlation between the wastewater surveillance time series and the incident COVID-19 cases time series. To do so we log-transformed (natural) quantifiable PCR results, assigning samples where SARS-CoV-2 RNA was not detected as zero and samples where SARS-CoV-2 RNA was detected below the limit of quantification as the midpoint between 0 and the limit of quantification (5 copies per mL). We then standardized log-transformed SARS-CoV-2 results by the log-transformed crAssphage results to come up with an intensity metric. Finally, we used the cross-correlation function to assess the correlation between the wastewater data and seven-day average of the clinical data using a Spearman’s correlation coefficient. We used three wastewater metrics to compare wastewater surveillance results with the 7-day rolling average of clinical cases at the school. First, we used log-transformed SARS-CoV-2 copies standardized to log-transformed crAssphage copies. Second, we used the log of SARS-CoV-2 gene copies. Third, we used non-transformed SARS-CoV-2 gene copies. We used R version 4.2.3 for all analyses and data visualizations [21].

### 2.6 Ethical approval

The study protocol was reviewed and approved by the Syracuse University Institutional Review Board (#23–414).

## 3. Results

### 3.1 Clinical cases

**Fig 1:**
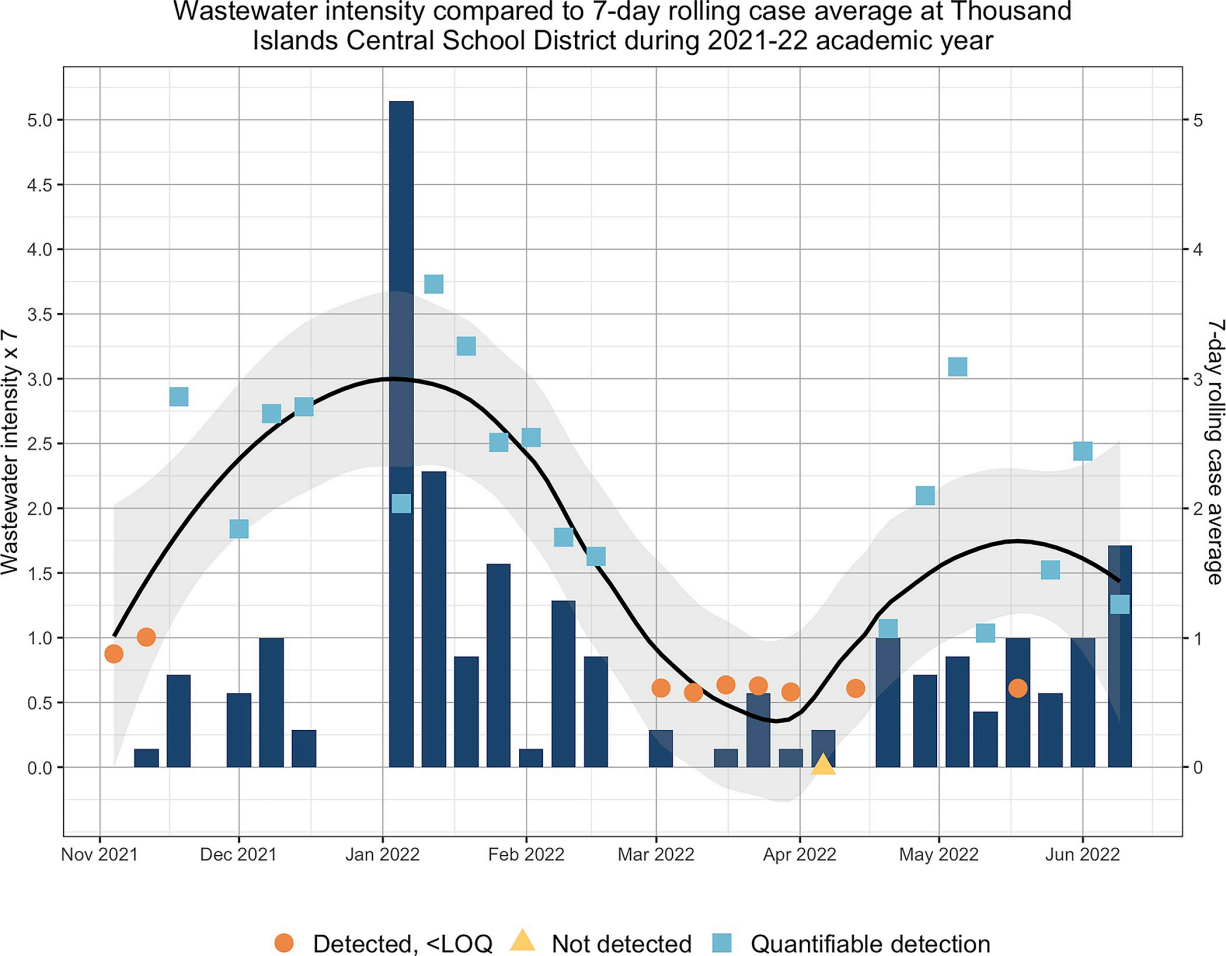
Weekly aggregated incident clinical case counts relative to wastewater testing during the 2021–22 academic year. The wastewater intensity is the log-transformed amount of SARS-CoV-2 in wastewater divided by the log-transformed amount of crAssphage in wastewater.

Between November 2021 and January 2022, incident clinical COVID-19 cases at TICSD reached a maximum of 5 cases per day. With the new year’s vacation came the greatest surge in cases, having 17 and 10 cases on January 3 and January 4, 2022, respectively. After January 4, 2022, the total weekly clinical cases declined, ranging from no weekly cases to 16 incident cases. It is worth mentioning that the period between the end of February and the beginning of April had the least number of incident cases reported, having no incident cases at all or a maximum of three in one day. That is unusual because a nine-day vacation preceded the end of February, and it had been apparent that the weeks after vacations exhibit spikes in new clinical cases. By the end of the 2021–2022 school year, a total of 179 cases were recorded by school administration (**Fig 1**).

### 3.2 Wastewater data

**Fig 2:**
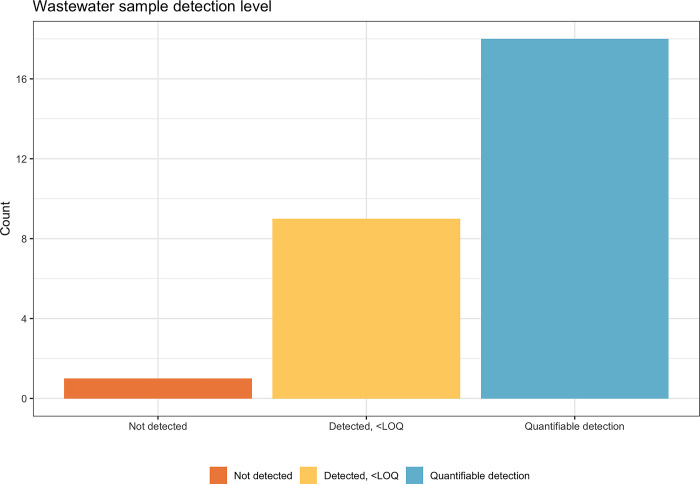
Characteristics of wastewater samples collected during 2021–2022 academic year. Not detected means no SARS-CoV-2 RNA was found by PCR. Detected, <LOQ means SARS-CoV-2 RNA was found but below the limit of quantification of 5 copies per mL.

During the school year, a total of 28 wastewater samples were collected by maintenance workers. A total of 9 (32%) of samples had detected levels of SARS-CoV-2 RNA and 18 (64%) contained quantifiable levels of SARS-CoV-2 RNA. Only one sample did not have detected or quantifiable levels of SARS-CoV-2 RNA (4%) (**Fig 2**). All wastewater samples were either collected on Wednesday (79%) or Thursday (21%) each week.

### 3.3 Correlation between SARS-CoV-2 RNA measured in wastewater and incident COVID-19 cases

**Fig 3:**
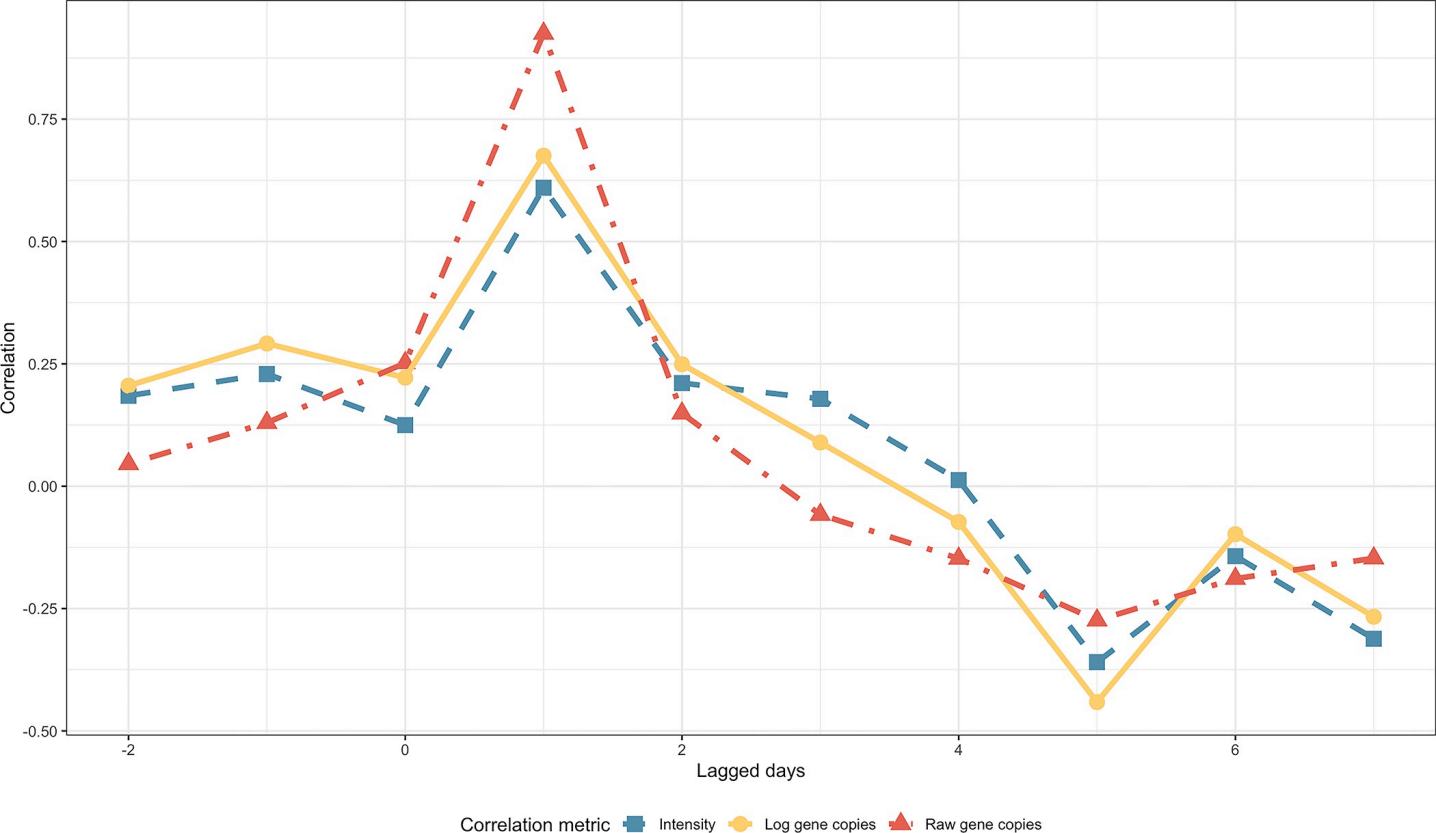
Correlation between clinical case 7-day roiling average and different wastewater metrics at TICSD. Intensity is defined as the log-transformed gene copies of SARS-CoV-2 divided by the log-transformed gene copies of crAssphage.

Wastewater levels best correlated with clinical cases the day after sample collection (**Fig 3**). Four days after sample collection, the correlation between wastewater levels of SARS-CoV-2 RNA and the number of clinical COVID-19 cases was zero. The only statistically significant correlations were observed for all three measures of SARS-CoV-2 in wastewater for a lead time of 1 day (p < 0.01 for all measures of wastewater). Raw gene copies of SARS-CoV-2 in wastewater had the highest correlation observed of 0.925, p < 0.001.

### 3.4 Sensitivity, specificity, positive and negative predictive values of wastewater surveillance

**Fig 4:**
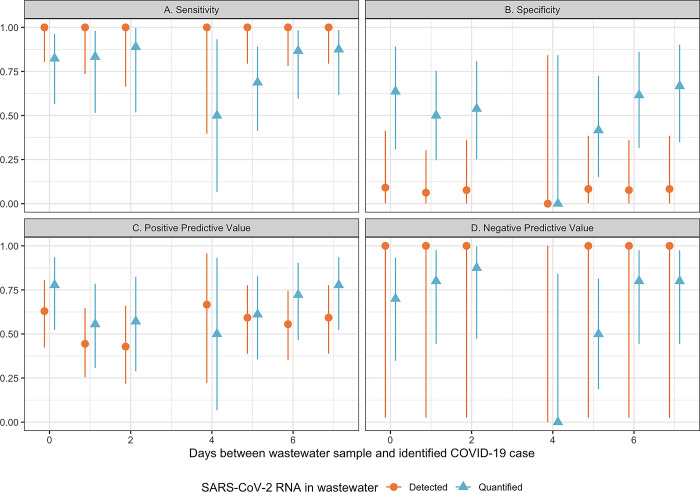
Sensitivity (**A**) to identify a COVID-19 case using wastewater surveillance at TICSD, specificity (**B**) the ability to identify the school building without a COVID-19 case using wastewater surveillance, positive predictive value (**C**) of a positive wastewater surveillance result to indicate a COVID-19 case among those at the school, and negative predictive value (**D**) of a negative wastewater surveillance result to indicate no COVID-19 cases among faculty, staff, and students at TICSD.

We observed a sensitivity of 100% (95% confidence interval [CI] = 80–100%) for wastewater surveillance to identify COVID-19 infections on the same day as a wastewater sample collection when considering the level of SARS-CoV-2 RNA was detectable. We observed a sensitivity of 82% (95% CI = 57–96%) when SARS-CoV-2 RNA in wastewater was quantifiable (**Fig 4A**). With more lead time, wastewater surveillance exhibited decreased sensitivity. When SARS-CoV-2 RNA was detectable, the sensitivity of wastewater was 100% (95% CI = 79–100%) for identification of COVID-19 cases up to 7 days out. Quantifiable levels of SARS-CoV-2 RNA in wastewater showed a sensitivity of 88% (95% CI = 62–98%) for identification of COVID-19 infections in TICSD up to a week out.

The PPV of wastewater correctly identifying a COVID-19 infection at the school on the same day of the sample collection was 63% (95% CI = 42–81%) for detected levels of SARS-CoV-2 RNA in the wastewater sample (**Fig 4C**). For quantifiable levels of SARS-CoV-2 RNA, the PPV of wastewater surveillance was 78% (95% CI = 52–94%). When considering a greater lead time, wastewater surveillance showed a PPV of 59% (95% CI = 39–78%) and 78% (95% CI = 52–94%) when SARS-CoV-2 RNA was detected and quantified in wastewater samples, respectively, at one week out.

We observed a specificity of 9% (95% CI = 0.2–41%) and 64% (95% CI = 31–89%) for wastewater surveillance to identify when the middle-high school did not have COVID-19 infections on the same day as wastewater sample collection when the SARS-CoV-2 RNA levels were detected and quantified, respectively (**Fig 4B**). When considering more lead time, wastewater surveillance showed a similar level of specificity for detected levels and slightly higher specificity for quantifiable levels of SARS-CoV-2 RNA in wastewater samples. Detected levels of SARS-CoV-2 RNA in wastewater samples displayed a specificity of 8% (95% CI = 0.2–38%) at 7 days out. Quantifiable levels of SARS-CoV-2 RNA had a specificity of 67% (95% CI = 35–90%) at 7 days out for identifying when the campus did not have COVID-19 infections.

The NPV of wastewater to correctly identify no COVID-19 infections at TICSD on the same day as wastewater testing, was 100% (95% CI = 3–100%) for detected levels of SARS-CoV-2 in wastewater and 70% (95% CI = 35–93%) for quantified levels (**Fig 4D**). When considering a greater lead time, wastewater surveillance showed a similar NPV for detected levels of SARS-CoV-2 RNA but was higher for quantifiable levels of SARS-CoV-2 RNA. At one week out, the NPV of wastewater correctly identifying the campus without COVID-19 infections was 100% (95% CI = 3–100%) for detected levels of SARS-2 RNA and 80% (95% CI = 44–97%) for quantifiable SARS-2 RNA in wastewater samples.

### 3.5 Comparison between Thousand Islands Central School and other Jefferson County catchment sites

**Fig 5:**
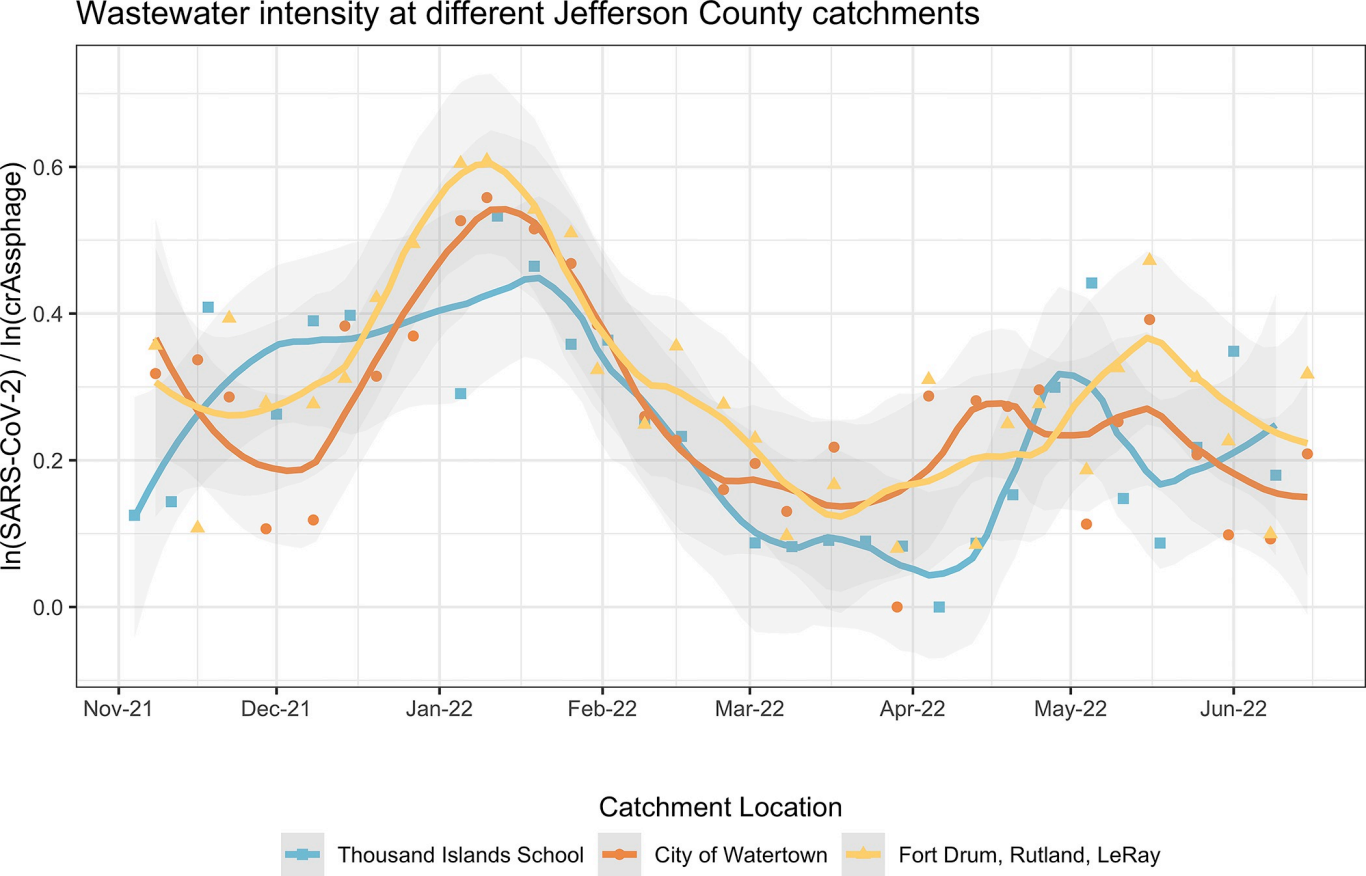
Comparison of SARS-CoV-2 intensity in wastewater at different catchment sites in Jefferson County.

During the time of this study, other locations in Jefferson County were also active participants in the New York State Wastewater Surveillance Network. Each catchment had one sample per week analyzed, similar to the middle-high school. There should be little overlap of the people surveilled between the catchments as they are roughly 30 minutes from each other. People in the City of Watertown or on Fort Drum do not attend school at Thousand Islands Central School; however school faculty or staff could come from either of those areas. A wastewater intensity comparison is shown (**Fig 5**). Overall, the increases and decreases in wastewater intensity match up well between the catchments for the City of Watertown and Fort Drum, Rutland, LeRay. The Thousand Islands Central School district also follows the same patterns of ebbs and flows in wastewater intensity as the other two catchment locations.

## 4. Discussion

We observed sensitivity and NPV above 0.75 and PPV above 0.5 for wastewater surveillance to correctly identify the presence or absence of COVID-19 cases in a middle and high school campus serving 600 students. The specificity of wastewater surveillance at the school was observed to be lower than the sensitivity, PPV, and NPV. The amount of SARS-CoV-2 RNA recovered in wastewater was highly correlated with the number of incident COVID-19 cases with a lead time of 1 day between wastewater sample collection and COVID-19 test date. Collectively, these results suggest that wastewater surveillance at the school-level may be a feasible approach to understanding SARS-CoV-2 transmission and risk within a school. However, the lead-time observed in this study demonstrated insufficient time to provide an early warning of SARS-CoV-2 transmission in a school before onward transmission from an index case can occur. Others have shown a greater lead time suggesting the possible use of wastewater surveillance as an early warning tool for the early stages of COVID-19 epidemics [22–25].

With an observed lead-time of only one day between sample collection date and COVID-19 test date, the ability of wastewater surveillance to act in an early warning capacity will depend highly on test seeking behavior. Even if the wastewater sample is analyzed the same day it is received, the sample must still be delivered to the laboratory after collection at the end of the school day. Thus, the information relayed from wastewater surveillance would arrive at the school the same day that someone would report a positive COVID-19 test, assuming the positive test is reported to the school the same day it is taken. If a school is conducting mass asymptomatic testing, wastewater surveillance may not add much benefit. However, wastewater surveillance is quite appealing in the absence of mass asymptomatic testing and particularly so given the comparative cost and logistics required.

The implications of the minimal lead-time are dependent upon the control strategies and COVID-19 policies in place. Interventions such as mass testing, social distancing, or school closure meant to disrupt transmission following detection in the wastewater would not prevent all transmission, as the wastewater test result is only communicated after an individual could transmit onward. Furthermore, it is not likely that every single infection at schools is found using wastewater surveillance, as detection requires toilet use on the school campus. Still, there is value in wastewater surveillance at the school level to ensure that any COVID-19 transmission is minimal, or that interventions such as using face masks are working [26]. Using this approach could potentially prevent unnecessary closures that were seen during the COVID-19 pandemic. School-level wastewater surveillance can also play a critical role in the community, serving as a sentinel surveillance site and providing data for subsequent clinical testing and vaccination campaigns in the community [27]. The trends of SARS-CoV-2 RNA in wastewater observed in the school matched very closely the trends observed in the community.

Improved understanding of SARS-CoV-2 transmission risk at the school level allows for more precise public health interventions if needed, potentially preventing unnecessary school closures. SARS-CoV-2 is not the only infectious disease that threatens the ability of schools to stay open. Before the COVID-19 pandemic, there have been school closures throughout the United States for various diseases including norovirus [28, 29], influenza [29–32] mononucleosis [33], pertussis [34], and even Ebola during the 2014–16 epidemic [35, 36]. Not all of these school closures may have been necessary. In fact, the school closure in Texas was due to unfounded fears about Ebola transmission–it occurred even without a transmission link between the suspected or confirmed cases and community members [35, 36]. The societal costs of school closures are immense, making it imperative to only utilize this nonpharmaceutical intervention when necessary. Wastewater surveillance is likely to work for most infectious diseases [37–39] and could be a potential tool to keep schools open during outbreaks.

Lower specificity in wastewater surveillance compared to clinical cases is expected for two reasons. First, wastewater surveillance captures more infections than clinical surveillance. In these instances the “false positives” identified are not really false, they just represent infections that were not found through clinical surveillance. Second, individuals shed SARS-CoV-2 into wastewater after they are no longer considered a clinical case. In these instances and in the absence of the first reason, the “false positives” identified would not indicate any infections missed by clinical cases.

Wastewater surveillance for COVID-19 has been scaled across the globe, including large-scale national programs throughout the European Union, in Australia and New Zealand, and smaller operations in many other countries [40]. Our results join other studies of school-based wastewater surveillance showing that measures of SARS-CoV-2 RNA in wastewater strongly correlates with clinical measures of disease [41–43]. These studies are all conducted in higher income countries, and the results are expected to be relevant wherever schools have piped sewer systems. In lower income countries, even access to sanitation at schools can be limited [44]. Schools in lower income countries are also an important location for infectious disease surveillance [45, 46]. But the logistics and feasibility of wastewater surveillance in schools without piped sewer systems needs to be better explored.

As we move beyond the COVID-19 emergency, wastewater surveillance in schools should be considered an essential component of schools’ emergency preparedness. All schools should have a plan to enact wastewater surveillance in the case of an emergency. This plan should include architectural drawings of sewer diagrams with key access points, contact information for laboratories that could test for novel, emerging pathogens, and plans and manuals on how to sample that school’s effluent wastewater potentially including schematics for building a sampler or schematics for passive sampling. In the case of a public health emergency, perhaps an emerging pathogen or a case of a vaccine-preventable pathogen such as measles or polio, the school’s emergency plan for wastewater surveillance could be quickly enacted to better inform the public health response and have closures guided by infectious disease surveillance rather than the imminent threat of infectious disease.

Support from the school and local health department is an essential factor in conducting wastewater surveillance. As part of the surveillance, we shared weekly memos with the local health department and the school administrators detailing and interpreting the results of the wastewater testing. This regular feedback and support for understanding the results of the efforts to do the testing is a key component of successful infectious disease surveillance programs. When considering the rollout of wastewater surveillance at the school level, each individual school participating should have epidemiologic support for the interpretation of wastewater testing results as pertains to that school, alongside the support for sample collection and analysis.

The high correlation of 0.75 between levels of SARS-CoV-2 in wastewater and incident clinical COVID-19 cases was observed during the Omicron surge of December 2021 –January 2022 and the BA2 surge later in April-May of 2022. Each new variant of SARS-CoV-2 presents with the same questions of whether the mutations have reduced the accuracy of our diagnostic tests or shedding rates differ. As to the first, we have observed no mutations that would threaten the accuracy of the IP2/IP4 primers that were used in this study, but others have observed decreased accuracy for the N2 primer and subsequently dropped it from analysis. We are not yet aware of longitudinal studies assessing fecal shedding rates with different variants, which would be valuable in understanding the results of wastewater surveillance. Although the correlation between levels of SARS-CoV-2 RNA in wastewater and incident COVID-19 cases has declined recently, we cannot ascertain how much if any of that decline is due to changes in fecal shedding due to the huge shift to at-home testing during the same time period.

A number of limitations are present in this study. Wastewater samples were only collected once weekly (Wednesday or Thursday), leaving a gap in surveillance activities throughout the weekend and early week. Also, clinical testing was not mandated but only available for individuals seeking a diagnostic test throughout the 2021–2022 school year. At no point did the school mandate testing for all students, faculty, and staff. Numerous asymptomatic infections are likely to have been missed in the clinical surveillance, however we assume that trends in asymptomatic transmission mirror the trends in clinical cases reported. Lastly, although wastewater samplers were homemade, there is no expectation that they would impact the results of this study [17].

## 5. Conclusions

Levels of SARS-CoV-2 RNA in a school’s wastewater correlated strongly with incident COVID-19 cases with a one-day lead time between sample collection dates. Wastewater surveillance at the school level has the ability to inform school administration and the public regarding risk of COVID-19 in that school. The short lead time would mean any reactive interventions would not necessarily prevent a generation of transmission. Still, implementing school-level wastewater surveillance would enable more precision public health intervention and all schools should have plans that would facilitate wastewater surveillance in a public health emergency.

## Supporting information

S1 Data Dataset used for analysis, including data dictionary. (XLSX)
